# Evaluation of lung adverse events with nivolumab using the spontaneous reporting system in Japan

**DOI:** 10.1038/s41598-023-35602-w

**Published:** 2023-05-31

**Authors:** Yuko Kanbayashi, Tadashi Shimizu, Asuka Kojima, Miku Anzai, Rika Kawai, Mayako Uchida

**Affiliations:** 1 Department of Education and Research Center for Clinical Pharmacy, Faculty of Pharmacy, Osaka Medical and Pharmaceutical University, 4-20-1 Nasahara, Takatsuki, Osaka 569-1094 Japan; 2 School of Pharmacy, Hyogo Medical University, 1-3-6 Minatojima, Kobe, Hyogo 650-8530 Japan; 3 Department of Education and Research Center for Pharmacy Practice, Faculty of Pharmaceutical Sciences, Doshisha Women’s College of Liberal Arts, 97-1, Kodominamihokotate, Kyotanabe-shi, Kyoto 610-0395 Japan

**Keywords:** Medical research, Oncology

## Abstract

This study was conducted to examine times to onset, incidence rates, and outcomes of nivolumab-induced lung adverse events (AEs), using the Japanese Adverse Drug Event Report database. We analysed data for the period between April 2004 and March 2021. Data on lung AEs were extracted, and relative risks of AEs were estimated using the reporting odds ratio. We analysed 5,273,115 reports and found 18,721 reports of nivolumab-related AEs, including 3084 lung AEs. Signals were detected for nine lung AEs: interstitial lung disease; pneumonitis; lung disorder; organising pneumonia; pleural effusion; pneumonia aspiration; pneumonia bacterial; radiation pneumonitis; and infectious pleural effusion. Among these, interstitial lung disease was the most frequently reported (68.7%) and included some fatal cases. A histogram of median times to onset showed AEs occurring from 34 to 79 days after the first dose, but some cases occurred even more than one year after starting administration. In conclusion, we focused on lung AEs caused by nivolumab as post-marketing AEs. Some cases could potentially involve serious outcomes, particularly in interstitial lung disease. Patients should be monitored for signs of the development of these AEs not only at the start of administration, but also over an extended time.

## Introduction

Nivolumab is a programmed death-1 (PD-1)-targeted monoclonal antibody, representing a revolutionary immunotherapy for cancer. This agent has been used in the treatment of various cancers, achieving improvements in overall survival^1–6^. In Japan, nivolumab has been approved for the treatment of advanced solid tumours, including malignant melanoma, non-small cell lung cancer, renal cell carcinoma, Hodgkin lymphoma, head and neck cancer, gastric cancer, malignant pleural mesothelioma, colorectal cancer, and oesophageal cancer. Nivolumab has been used in combination with chemotherapy and has been reported to prolong both overall and progression-free survival in clinical trials^2–6^. However, nivolumab can cause unique immune-related adverse events (irAEs). Among these, interstitial lung disease is the most common serious adverse event (AE), representing a rare but potentially fatal irAE necessitating nivolumab discontinuation. Other irAEs include adrenal insufficiency, hypopituitarism, colitis/severe diarrhoea, eye diseases, haematological disorders, hepatitis, thyroid dysfunction, myasthenia gravis, myocarditis, nephritis/renal dysfunction, rash, and type 1 diabetes mellitus^7–11^. However, inadequate management of AEs may force discontinuation of nivolumab treatment until the events can be controlled, which may in turn incur disadvantages to the patient, such as decreased efficacy. In other words, the life prognosis of the patient is also affected. Bukamur et al. reported that rapid identification of lung-related toxicities and appropriate treatment of patients can prevent morbidity and mortality in cancer patients treated with immune checkpoint inhibitors (ICIs)^12^. In addition, although nivolumab has been widely used in patients since its launch, detailed information on lung-specific AEs from post-marketing monitoring has not been reported^13^. We therefore conducted this study to examine times to onset, incidence rates, and outcomes of nivolumab-induced lung AEs in patients with cancer. Analyses in this study were based on information obtained from the spontaneous reporting system in the Japanese Adverse Drug Event Report database (JADER), a large pharmacovigilance database provided by the Pharmaceuticals and Medical Devices Agency (PMDA). The identification for safety signals of chemotherapeutic agents using the spontaneous adverse event reporting databases could provide a useful method for generating hypotheses about possible drug-drug interactions with unknown or potential AEs.

## Results

### Incidence of lung AEs with nivolumab

We used ID numbers to link three tables: DRUG (3,875,874 reports); REAC (1,096,193 reports); and DEMO (693,295 patients). Duplicate data were deleted from the DRUG and REAC databases^14^. AEs are caused by three types of factors: "suspected drugs," "concomitant drugs," and "interaction". These terms are all used to describe medications that are taken together. All data in the category of "suspected drugs" were retrieved and used as the basis for the study "table of data" (1,772,494 reports).

**Figure 1:**
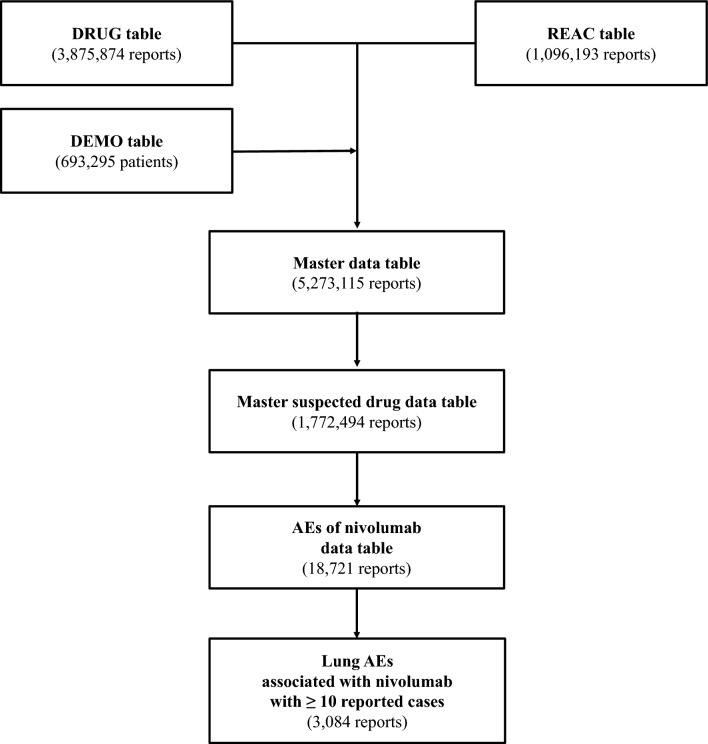
Process of constructing a data analysis table.

On reviewing this data table, we found 18,721 reports of nivolumab-related AEs, including 3084 lung AEs (Fig. 1). Table 1 lists the patient characteristics. Males made up approximately 78.3% of patients. Lung AEs were most common among patients in their 70 s (37.6%), followed by those in their 60 s, according to the age distribution of the study group (35.0%). We found that the following numbers of cases have been reported for concomitant use of anticancer drugs that may cause lung AEs such as T-lymphocyte antigen-4 (CTLA-4) inhibitors or molecular targeted drugs such as epidermal growth factor receptor-tyrosine kinase inhibitors (EGFR-TKI): ipilimumab (32 cases), osimertinib (4 cases), and gefitinib (3 cases). Among cases of nivolumab-induced lung AEs, the following numbers of patients had major pre-existing pulmonary diseases: 1489 with lung cancer, 780 with lung metastases, 180 with pleural metastases, 143 with chronic obstructive pulmonary disease, 119 with emphysema, 41 with asthma, 29 with atelectasis, and 29 with pleural mesothelioma and so on.

**Table 1:** Characteristics of patients exhibiting lung adverse events related to nivolumab.

Variable	Value (%)
Number of patients	3,084
Sex	
Male	2,416 (78.3)
Female	623 (20.2)
Unknown	45 (1.5)
Age (years)	
≤ 10 s	1 (0.03)
20 s	6 (0.2)
30 s	20 (0.6)
40 s	121 (3.9)
50 s	354 (11.5)
60 s	1,079 (35.0)
70 s	1,161 (37.6)
80 s	250 (8.1)
90 s	3 (0.1)
Unknown	89 (2.9)

Among the types of lung AEs caused by nivolumab, reported numbers of interstitial lung disease, pneumonitis, lung disorder, organising pneumonia, pleural effusion, pneumonia aspiration, pneumonia bacterial, *Pneumocystis jirovecii* pneumonia, dyspnoea, and radiation pneumonitis associated with nivolumab were 2,120, 186, 185, 123, 85, 63, 58, 40, 40, and 39 respectively (Table 2). RORs with a lower limit of the 95%CI > 1 were interstitial lung disease, pneumonitis, lung disorder, organising pneumonia, pleural effusion, pneumonia aspiration, pneumonia bacterial, radiation pneumonitis, and infectious pleural effusion, with values of 4.56 (95%CI 4.35–4.78), 6.38 (5.49–7.40), 5.36 (4.62–6.22), 8.17 (6.79–9.83), 2.10 (1.69–2.60), 1.44 (1.12–1.85), 1.80 (1.39–2.33), 7.84 (5.65–10.87) and 1.84 (1.06–3.19), respectively. Signals were detected for nine (interstitial lung disease, pneumonitis, lung disorder, organising pneumonia, pleural effusion, pneumonia aspiration, pneumonia bacterial, radiation pneumonitis, and infectious pleural effusion) out of the ten lung AEs.

**Table 2:** Numbers of reports and RORs of lung adverse events related to nivolumab.

Adverse events	Cases (n)	Non-cases (n)	Rate (%)	ROR	95%CI	*p*-value
Interstitial lung disease	2,120	1,705,996	11.32	4.56	4.35–4.78	< *0.001*
Pneumonitis	186	1,751,018	0.99	6.38	5.49–7.40	< *0.001*
Lung disorder	185	1,750,512	0.99	5.36	4.62–6.22	< *0.001*
Organising pneumonia	123	1,752,354	0.66	8.17	6.79–9.83	< *0.001*
Pleural effusion	85	1,749,967	0.45	2.10	1.69–2.60	< *0.001*
Pneumonia aspiration	63	1,749,679	0.34	1.44	1.12–1.85	*0.006*
Pneumonia bacterial	58	1,750,750	0.31	1.80	1.39–2.33	< *0.001*
*Pneumocystis jirovecii* pneumonia	40	1,746,540	0.21	0.52	0.38–0.71	< *0.001*
Dyspnoea	40	1,747,285	0.21	0.58	0.42–0.79	< *0.001*
Radiation pneumonitis	39	1,753,306	0.21	7.84	5.65–10.87	< *0.001*
Respiratory failure	34	1,749,819	0.18	0.81	0.57–1.13	0.244
Pulmonary embolism	33	1,749,890	0.18	0.80	0.56–1.12	0.210
Acute respiratory distress syndrome	15	1,751,686	0.08	0.67	0.40–1.12	0.135
Bronchopulmonary aspergillosis	14	1,751,964	0.07	0.72	0.43–1.23	0.300
Infectious pleural effusion	13	1,753,112	0.07	1.84	1.06–3.19	*0.036*
Pulmonary alveolar haemorrhage	13	1,751,906	0.07	0.65	0.38–1.13	0.141
Eosinophilic pneumonia	12	1,752,310	0.06	0.77	0.44–1.36	0.444
Cardio-respiratory arrest	11	1,750,964	0.06	0.37	0.20–0.66	< *0.001*

“Cases” indicates the number of reported cases of lung AEs. ROR: reporting odds ratio; 95%CI: 95% confidence interval. Italicized *p*-values represent statistically significant results. We used ≥ 10 reports for each type of lung AE. All analysed data were obtained from the Japanese Adverse Drug Event Report database. Hypothesis tests were two-sided, with statistical significance set at p < 0.05. *P*-values were calculated using Fisher’s exact test.

### Time to onset of lung AEs with nivolumab

**Figure 2:**
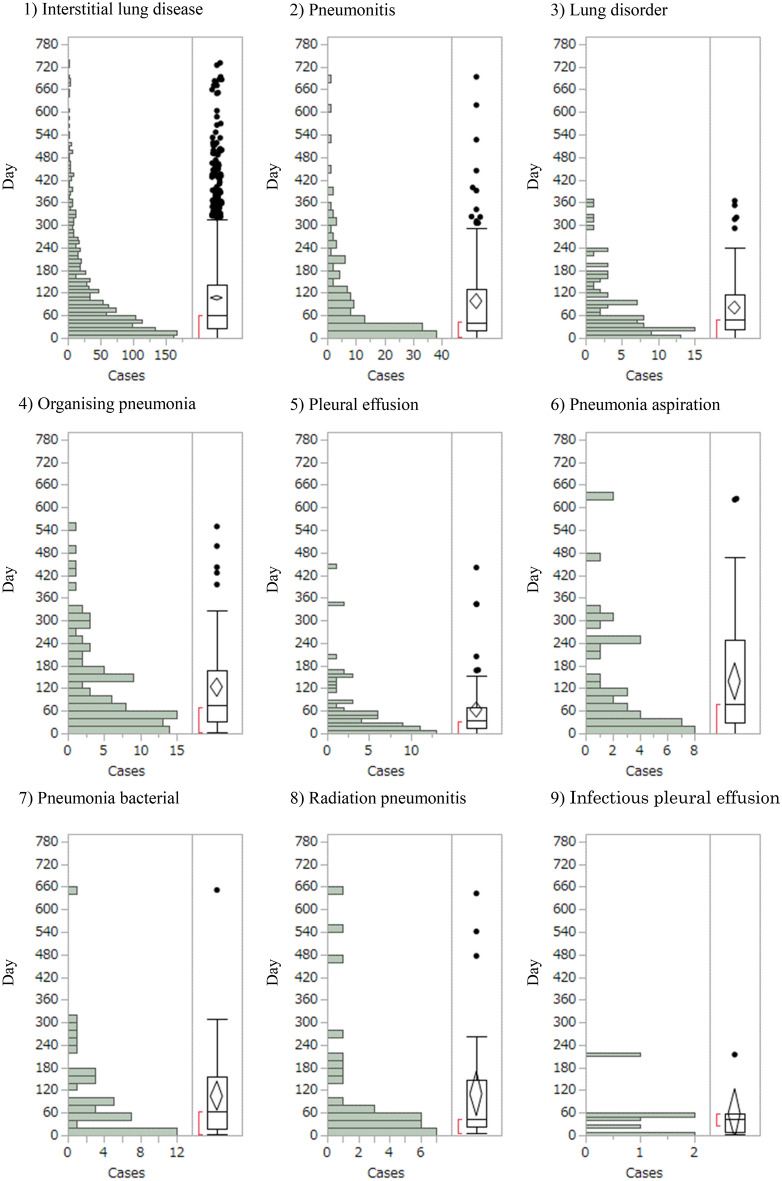
Histogram of lung adverse events for: 1) interstitial lung disease; 2) pneumonitis; 3) lung disorder; 4) organising pneumonia; 5) pleural effusion; 6) pneumonia aspiration; 7) pneumonia bacterial; 8) radiation pneumonitis; 9) infectious pleural effusion.

A histogram of the time to onset of the nine detected lung AE signals showed that these events occurred 34–79 days after starting nivolumab administration (Fig. 2). Median time (days [range]) to onset [interquartile range (IQR)] for interstitial lung disease, pneumonitis, lung disorder, organising pneumonia, pleural effusion, pneumonia aspiration, pneumonia bacterial, radiation pneumonitis, and infectious pleural effusion caused by nivolumab were 62 (26–142), 42 (20–131), 49 (24–112), 75 (34–169), 34 (15–70), 79 (30–249), 64 (17–154), 44 (23–148), and 43 (8–59), respectively. The Weibull distribution of the histogram for the time to onset showed that the range of 95%CIs for shape parameter β were < 1 for interstitial lung disease, pneumonitis, pleural effusion and pneumonia aspiration, and approximately 1 for other AEs (Table 3).

**Table 3:** Median timings and Weibull parameters of lung adverse events.

Adverse events	Cases (n)	Median (days) (quartiles, 25–75%)	Scale parameter	Shape parameter
			α (95%CI)	β (95%CI)
Interstitial lung disease	1,572	62 (26–142)	102.32 (96.55–108.39)	0.90 (0.87–0.94)
Re-administration	15	129 (74–248)	181.80 (104.75–306.37)	1.06 (0.68–1.52)
Other	1557	62 (26–141)	101.67 (95.91–107.73)	0.90 (0.87–0.94)
Pneumonitis	149	42 (20–131)	89.20 (72.80–108.73)	0.85 (0.75–0.96)
Re-administration	3	41 (32–59)	47.91 (31.80–71.84)	4.26 (1.38–9.18)
Other	146	42 (19–131)	89.92 (73.16–109.95)	0.85 (0.75–0.96)
Lung disorder	101	49 (24–112)	78.07 (62.16–97.39)	0.92 (0.79–1.07)
Re-administration	1	50 (50–50)	–	–
Other	100	48 (23–116)	78.20 (62.13–97.76)	0.92 (0.78–1.07)
Organising pneumonia	98	75 (34–169)	124.65 (101.06–152.74)	1.02 (0.87–1.19)
Re-administration	1	58 (58–58)	–	–
Other	97	80 (34–169)	125.22 (101.37–153.67)	1.02 (0.87–1.19)
Pleural effusion	68	34 (15–70)	55.83 (40.51–76.12)	0.81 (0.67–0.96)
Pneumonia aspiration	42	79 (30–249)	122.66 (80.10–184.42)	0.78 (0.60–0.99)
Pneumonia bacterial	41	64 (17–154)	97.24 (65.78–141.36)	0.86 (0.67–1.08)
Re-administration	1	46 (46–46)	–	–
Other	40	66 (17–156)	98.25 (65.92–143.90)	0,86 (0.66–1.08)
Radiation pneumonitis	31	44 (23–148)	95.99 (58.82–153.09)	0.80 (0.61–1.02)
Infectious pleural effusion	7	43 (8–59)	54.28 (18.46–149.88)	0.87 (0.44–1.44)

“Cases” indicate the number of reported cases of lung AEs. 95%CI: 95% confidence interval. Detected lung AE signals were analysed to determine the time to onset.

### Outcomes after AE occurrence

**Figure 3:**
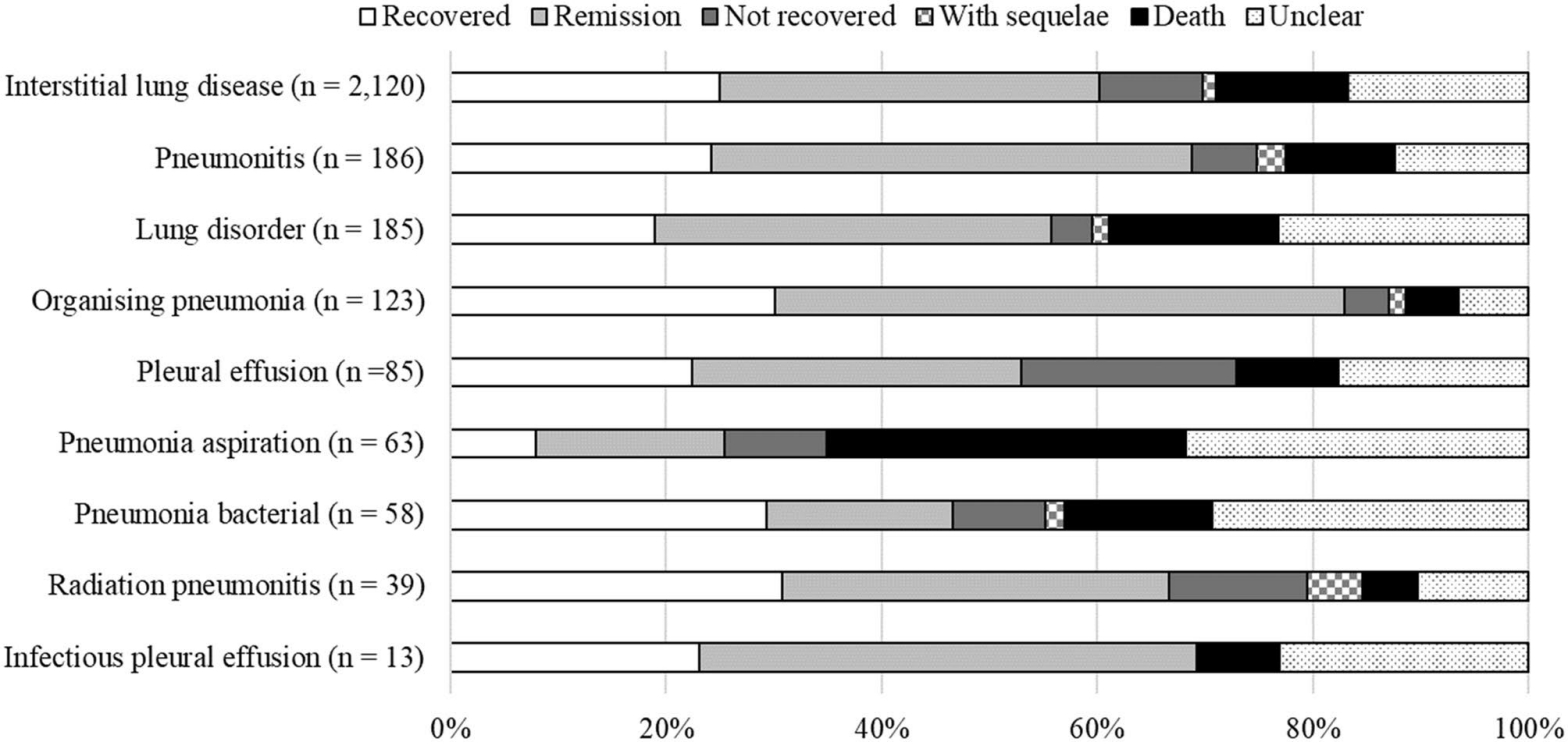
Percentage of nine AEs associated with nivolumab by outcome.

Percentages of outcomes after commencement of the nine AEs (recovery, remission, not recovered, recovered with sequelae, death, and unclear) are presented in Fig. 3. In all nine AEs, fatal results were observed.

## Discussion

This study focused on post-marketing nivolumab-induced lung AEs and suggested that those AEs for which signals were detected were interstitial lung disease, pneumonitis, lung disorder, organising pneumonia, pleural effusion, pneumonia aspiration, pneumonia bacterial, radiation pneumonitis, and infectious pleural effusion.

Among these, interstitial lung disease was the most frequently reported (68.7%, 2120/3084), with fatal cases also reported. Interstitial lung disease caused by nivolumab has been reported in clinical trials^15–18^, and the present results are consistent with the clinical findings. On the other hand, the Weibull distribution showed that interstitial lung disease developed early after nivolumab administration. The median time of onset for interstitial lung disease in this study was 62 days (IQR, 26–142 days). This represented an earlier onset than shown in the results of clinical trials (median time of onset, 88.5 days)^15–19^. Clinicians should thus pay close attention to the onset of interstitial lung disease right after the start of nivolumab administration. In previous studies, interstitial lung disease has shown a high mortality rate if left untreated, and early diagnosis and treatment are known to significantly reduce mortality^20^. AE data obtained in clinical trials before a drug is approved represent information obtained from a relatively small population under a variety of constraints. In contrast, after approval, new trends in AEs that may occur may be discovered through the repeated use of the drug in patients with various characteristics. Pharmacovigilance to monitor drug safety is therefore important for the proper use of all drugs^21–23^. In this study, although the incidence of interstitial lung disease did not increase in a dose-dependent manner, continuous monitoring is recommended throughout the entire treatment period, as several cases were observed after almost 2 years of treatment. Since the interstitial lung disease caused by nivolumab is frequently associated with fatal outcomes, clinicians should pay close attention to its development not only at the start of administration, but also over an extended period.

Fatal outcomes were observed in all nine AEs for which signals were detected. Of these, organising pneumonia occurred at a constant rate, so continuous monitoring is recommended throughout the entire treatment period. Here, the most common interstitial lung disease associated with ICIs is organising pneumonia with lymphocytic infiltration. In general, organising pneumonia is more responsive to steroid therapy and shows better prognosis^16^. The immediate diagnosis and treatment of irAEs is thus crucial for achieving good outcomes.

All other AEs for which signals were detected occurred relatively early after nivolumab administration. Among the reported cases, a histogram of median time to onset showed that events occurred from 34 to 79 days after the first nivolumab administration. In clinical trials, AEs tended to develop later after administration^15–19^, but the accumulation of cases from actual clinical practice has clarified that AEs develop earlier after nivolumab administration. Clinicians should be alert to the development of these AEs, particularly in the initial stages of nivolumab administration. On the other hand, as a result of enhanced safety monitoring (stimulated reporting) during the post-marketing surveillance, which is imposed on almost all new drugs as well as the drug use-results survey, the ratio of reports per exposure during the first six months after market launch tends to be higher than in subsequent periods. This may be one of the reasons why all AEs occurred relatively early after nivolumab administration. Furthermore, interstitial lung disease, pneumonitis, pleural effusion, pneumonia aspiration, pneumonia bacterial, and radiation pneumonitis can still arise more than 1 year after first administration. Consistent monitoring over the course of the administration is therefore warranted.

Fatal cases of infection-related lung AEs were also reported for pneumonia bacterial and infectious pleural effusion. Since infections have been reported to occur early after nivolumab administration and fatal cases have been seen, clinicians should be alert for the onset of such infections immediately after administration. Lombardi et al. reported the most frequently reported infections involving patients treated with ICIs involved the respiratory tract (including nasopharyngitis, upper respiratory tract infections, and pneumonia) and the urinary tract^24^. Unlike cytotoxic chemotherapy, bone marrow suppression does not occur, so "neutropenia" is not a problem. On the other hand, the situation changes drastically when irAEs occur. In other words, as in the present study, steroids and infliximab, an antitumour necrosis factor-alpha antibody, are often administered for irAEs. Naturally, the risk of infection due to "cellular immunodeficiency" caused by these drugs is increased. Ross et al. also reported infectious complications are encountered with ICIs and correlate with steroid use^25^. Thus, differentiation of diseases is important because there is an important clinical difference between anti-inflammatory/immunosuppressive treatment due to irAE and treatment for infection. Further research is needed to delineate between the incidence of irAEs and infections. Although the risk of infectious complications and exacerbations may be lower than with conventional cytotoxic anticancer agents, the immune pathways modified by ICIs may vary depending on the host condition, and individual case studies are necessary.

The present results have to be considered in light of some limitations. First, the JADER is based on self-reported data, which may contribute reporting biases such as over- and underreporting. That is, JADER’s information sources are mainly spontaneous reports, such as individual case reports from companies, research reports, and direct reports from medical institutions. In addition, serious AEs from use-results surveys (including all-case surveillance) and post-marketing clinical trials are also included. In the case of spontaneous reports, there is no “denominator” to calculate the incidence rate, and the information collected after marketing may include underreporting and other reporting biases due to advances in diagnosis and treatment, media influence, and other factors. Second, the lack of comprehensive medical records and medication histories limits the scope of the analyses, as dosages, durations, clinical laboratory data, severity of AEs, medical history and more information on concomitant medications of nivolumab use were unavailable. Third, the majority of cases involved men (78.3%) and the most common age groups affected were the 70 s (37.6%) and 60 s (35.0%). Thus, male sex and higher age group cannot be ruled out as contributing factors in lung AEs. Fourth, we could not exclude the possibility that AEs may have been caused by concomitant use of anticancer drugs, such as CTLA-4 inhibitors (e.g. ipilimumab) or molecular targeted drugs such as EGFR-TKI (e.g. gefitinib, osimertinib). The lack of data on medication history limits our ability to draw definite conclusions regarding the relationship between these drugs and the occurrence of lung AEs in nivolumab-treated patients. Fifth, potential confounding, selection, and information biases cannot be fully excluded from this study. Almost all new drugs (including those with additional indications) are subject to drug use-results surveys, and all-case surveillance is conducted for some anticancer drugs in Japan. However, in addition to unsolicited reports, there is a quality problem that a considerable amount of information from unsolicited safety reports based on requests is included. Therefore, JADER is considered to have a qualitative problem in that it includes a considerable amount of information from involuntary safety reports in addition to voluntary reports. However, the results of this study were based on extracted data in which nivolumab was judged to be the suspect drug by a reporter (physician or pharmacist) who knew the details of the clinical course. Our report provides useful information for monitoring lung AEs associated with nivolumab.

In conclusion, we focused on lung AEs caused by nivolumab as post-marketing AEs. Interstitial lung disease, pneumonitis, lung disorder, organising pneumonia, pleural effusion, pneumonia aspiration, pneumonia bacterial, radiation pneumonitis, and infectious pleural effusion could potentially result in serious outcomes after administration of nivolumab. In particular, interstitial lung disease occurred in large numbers and over a long period of time. Some other AEs developed not only at the start of administration, but also over the long term. Patients should be monitored for signs of the development of these AEs for a long period after starting nivolumab administration.

## Methods

### Data source

We used data from JADER public releases^26–29^. This database, which includes AE cases, is available for free download from the PMDA website (https://www.pmda.go.jp/english/index.html, https://www.info.pmda.go.jp/fukusayoudb/CsvDownload.jsp). We examined AE reports for the period between April 2004 and March 2021. The data structure of the JADER is made up of four different datasets: patient demographics (DEMO); drug information (DRUG); AE (REAC); and medical history (HIST). AEs in the JADER are coded according to the terminology preferred by the Medical Dictionary for Regulatory Activities/Japanese version 24.1 (www.pmrj.jp/jmo/php/indexj.php).

We first removed duplicate cases from the DRUG and REAC tables, referencing a previous study^14^. Related case data from the DRUG, REAC, and DEMO tables were then merged using the identification number of each AE case. Medication contributions to AEs were classified as “suspected medicine,” “concomitant medicine,” or “interaction.” We only extracted those cases showing a contribution classified as a “suspected medicine”.

We used the JADER, which contains spontaneous AE reports filed to the PMDA, to examine links between nivolumab and lung AEs. Each lung-specific AE coded according to the terminology recommended by the Medical Dictionary for Regulatory Activities was collectively referred to as a lung AE in this study.

### Statistical analyses

The relative risk of each AE was calculated using the reporting odds ratio (ROR) and data for lung AEs with ≥ 10 reported instances. ROR is frequently used in the spontaneous reporting database as an indicator of the relative risk of an AE. We used the analysis data table and constructed 2 × 2 tables based on two classifications: presence or absence of the “lung AE”; and presence or absence of suspected nivolumab use. The ROR was calculated by dividing the reported rate of the AE attributable to nivolumab by the reported rate of the same AE attributable to all other drugs in the database. The signal of an AE was considered positive if the lower limit of the 95% confidence interval (CI) of the ROR was > 1^30^.

For reports in which the date of onset of an AE, date of start of administration, and date of end of administration were described as year/month/day or year/month^14^, onset time was calculated as "(onset date of AE)—(administration start date) + 0.5" in principle^31^. If there was a period of non-administration for > 1 year, the date of first administration of the most recent continuous administration period was used. The time to onset of AEs for analysis was limited to 2 years (730 days). Scale parameter α and shape parameter β were used to represent the Weibull distribution. The scale parameter α represents the scale of the distribution function, as the quantile in which 63.2% of AEs occur^32^. A large value for the scale indicates a wide distribution, while a small value indicates a narrow distribution. The shape parameter β represents the change in hazard over time in the absence of a reference population. An upper limit of the 95%CI of the β value < 1 indicates that the hazard increases initially, then decreases (early failure type). A β value of or around 1 with the 95%CI containing 1 indicates that the hazard remains constant throughout the exposure period (random failure type). A lower limit of the 95%CI for β > 1 indicates that the hazard increases over time (wear-out failure type).

### Ethical approval

Ethics approval was not sought for this study; given the database-related, observational design without direct involvement of any research subjects. All results were obtained from data openly available online from the PMDA website (https://www.pmda.go.jp/english/index.html, https://www.info.pmda.go.jp/fukusayoudb/CsvDownload.jsp). All data from the JADER database were fully anonymized by the relevant regulatory authority before we accessed them. Thus, all methods were performed in accordance with the relevant guidelines and regulations.

## Data Availability

The datasets generated and analyzed during the current study are available from the corresponding author on reasonable request.
